# Common and Differing Elements Across Caregiver-Mediated Social Communication Programs Supporting Autistic Children: A Scoping Review With Content Analysis

**DOI:** 10.1177/13623613261469913

**Published:** 2026-08-12

**Authors:** Vipula Rajesh Kumar, Lauren Choi, Janis Oram, Amanda Binns

**Affiliations:** 1 Western University, London, Ontario, Canada; 2 University of Toronto, Ontario, Canada

**Keywords:** autism, caregiver-mediated interventions, social communication, coaching strategies, program elements, content analysis

## Abstract

Caregiver-mediated interventions are widely used to support social communication development in young autistic children. While many such interventions are evidence-informed, limited information exists about their shared and unique elements, making it challenging for caregivers and clinicians to tailor program selection to individual needs and preferences—an essential part of personalized care in autism. This scoping review with content analysis examined 18 commercially available, caregiver-mediated social communication interventions to identify similarities and differences in child skills targeted and caregiver-coached strategies. Data were extracted from program manuals, fidelity tools, published literature, and program websites for eight developmental social pragmatic (DSP) programs and 10 naturalistic developmental behavioral interventions (NDBIs). Programs varied in scope, with some supporting a broad range of child skills and others focusing on specific areas. Across all programs, 70 caregiver-coached strategies were identified, and then thematically grouped into eight categories aligned with the developmental skills the strategies aim to support. Although some strategies were common across all programs, some unique strategies were also identified. In addition, there were notable differences in how these strategies were operationalized despite using similar terminology, underscoring the need for more precise, clearly defined fidelity measures to enable accurate assessment of caregiver implementation and meaningful comparisons across programs. This study lays the groundwork for future research to identify active ingredients of interventions and supports more informed, individualized decision-making in clinical practice.

**Lay Abstract:**

Young autistic children may engage with others and communicate in ways that differ from non-autistic peers, and may need support to develop skills to communicate with, and navigate interactions with others. Many programs have been developed for this purpose, with a growing number of programs including caregivers in this process. In programs where caregivers play a primary role, professionals coach them to use certain techniques that can support social communication. Although many such programs exist, we still lack clarity on how these programs are similar or different, making it tough for families to choose among them. To address this gap, we reviewed 18 well-known caregiver-led programs to compare the skills they aim to support children with, and the specific techniques they teach caregivers. We gathered information from program resource materials, published research, and official program websites. We found that some programs focused on supporting a wide range of skills, whereas others targeted only certain developmental areas. In total, we identified 70 distinct caregiver-coached techniques, which we organized into eight categories. Many techniques were common across all programs, but others were unique to specific programs. Even among the shared techniques, there were differences in the way they were practiced, despite using the same strategy name. For example, a technique like “following the child’s lead” could look quite different depending on the program. These findings show that having descriptions of how techniques should be used is important to understanding and comparing different programs. Overall, these findings provide families with information about program ingredients and can be used to guide clinicians toward programs that match individuals’ needs and wants. This work also paves way for future research exploring how these social communication programs work, and evaluating which techniques are most effective for specific groups of children and families.

## Introduction

Family-centered practices are a hallmark of early support for autistic children (Wong et al., 2015). These involve training and coaching primary caregivers, enabling them to transfer the strategies learned in a therapeutic setting to naturally occurring daily social situations (Shire et al., 2015). Development of social communication skills is a key priority area for caregivers of autistic preschoolers (Pituch et al., 2011). In young children, early social communication integrates a range of foundational skills including *joint attention* (the reciprocal triadic attention between a caregiver, child and an event/object through shared attention/social orienting), *imitation, shared referencing* (e.g., seeking social information from others following an unexpected or ambiguous event), and *joint engagement* (i.e., reciprocal interactions between the caregiver and the child which may include skills such as imitation, turn taking, etc.) (Hansen et al., 2018; Warreyn et al., 2014). These early social communication skills have been hypothesized to support and predict advanced developmental trajectories (see Gulsrud et al., 2014; Lobban-Shymko et al., 2017; Morgan et al., 2020; Rautakoski et al., 2021).

Research has shown that caregivers can be successfully taught to use social communication strategies (e.g., responsiveness, reduction in caregiver directiveness) through interventions such as *Joint Attention Symbolic Play Engagement and Regulation* (JASPER; e.g., Kasari et al., 2010), *More Than Words* (MTW; e.g., Girolametto et al., 2007), and *Enhanced Milieu Teaching* (e.g., Hancock & Kaiser, 2002). Furthermore, a range of caregiver-mediated interventions have demonstrated improvements in children’s communication as well as increased caregiver confidence in supporting their child (Barton & Fettig, 2013; Brookman-Frazee et al., 2006; Kaiser & Trent, 2007; McConachie & Diggle, 2006; Roberts & Kaiser, 2011).

Although several evidence-informed, caregiver-mediated interventions are available to support the development of social communication and related skills in young autistic children, our understanding of similarities and differences across these programs remains incomplete. This lack of clarity makes it difficult to associate improvements in children’s skills with specific strategies and challenges decision-making for caregivers and clinicians in seeking programs that best align with the needs, preferences, and values of each child and family.

Frost et al. (2020) addressed this gap for naturalistic developmental behavioral interventions (NDBIs), which share a common theoretical basis but nonetheless have variations across core intervention elements. They developed a taxonomy of common elements along with a new rating scale to assess implementation fidelity. This taxonomy represents a first step toward future identification of the active or most critical ingredients that bring about change within NDBIs. Inspired by the work of Frost et al. (2020), we aimed to bridge this knowledge gap about caregiver-mediated social communication programs supporting autistic preschoolers and their caregivers. Understanding similarities and differences across programs’ intervention elements will help the clinical and scientific communities gain better clarity on *how* these programs are delivered. It will also lay the groundwork for designing future research aimed at determining the most essential components of these interventions, thereby aligning with calls for autism support service research to explore program moderators and mediators (Klinger et al., 2021; Schreibman et al., 2011). Given the heterogeneity in treatment response, it is important to identify specific factors that account for this variability to support personalization of treatment options for autistic children and families, and to determine *a priori* which interventions may be most effective for a subgroup of children, aligning with the field’s shift toward individualized care rather than a one-size-fits-all approach (Schreibman et al., 2011). Accordingly, a critical first step is to characterize how these programs are similar and how they differ. Therefore, our research questions were:

1. What are the similarities and differences in specific child skill development areas targeted across commercially available, evidence-informed, caregiver-mediated social communication programs for young autistic children?
2. What are the similarities and differences in caregiver-coached strategies across these programs?

## Methods

Our study protocol was registered with OSF Registries (10.17605/OSF.IO/R528M) and adheres to the Preferred Reporting Items for Systematic Reviews and Meta-Analyses Extension for Scoping Reviews (PRISMA-SCR; Tricco et al., 2018), as detailed in Supplemental Appendix A. Protocol deviations are reported in Supplemental Appendix B.

### Phase 1

A search strategy was developed in consultation with an expert health scientist to generate a list of evidence-informed and commercialized social communication interventions for young autistic children (see Supplemental Appendix C for search queries). Studies published between January 2010 and May 2023 were searched on seven databases (Scopus, ERIC, PsycINFO, EMBASE, AMED, PubMed and CINAHL). Consistent with the domains of social communication described by Adams (2005) and the American Speech-Language-Hearing Association (ASHA, n.d.), programs needed to include supporting areas of *social interaction* (recognition of others as social beings and intersubjectivity), *social cognition* (e.g., theory of mind), *pragmatics* (contextual use of spoken and non-spoken communication), and *language processing* (decoding and encoding of grammatical structures, word meanings, and phonological forms).

The following inclusion criteria were used for Phase 1: (a) child participants were between 1 month and 5 years, 11 months, or the mean age of the group was below 6 years at the time of data collection; (b) children were diagnosed with autism (inclusive of past diagnostic labels PDD-NOS or Asperger syndrome) or were suspected to have autism; (c) the study was a RCT that evaluated the effectiveness of a caregiver-mediated social communication intervention program; (d) the intervention program is commercialized and currently offered; (e) the study demonstrated program effectiveness or efficacy for at least one child or caregiver outcome; and (f) the article was written in English.

Search results were then imported into Covidence systematic review software (2023). Two reviewers independently screened the titles and abstracts and reviewed the full texts, achieving >95% inter-rater reliability on an initial 25% of articles. The first reviewer then independently reviewed the remaining articles. A third reviewer was consulted to resolve any disagreements.

### Phase 2

For each program identified in Phase 1, we gathered all possible resources that could provide data for content analysis on intervention elements (i.e., program manuals, coaching guides, fidelity tools, textbooks, articles, and official program websites). We also contacted the respective developers and/or lead contact persons of each program to procure any of the above resources that were not publicly available.

### Data Extraction

Data were extracted by two researchers independently from the resource materials obtained in Phase 2. To support foundational understanding of each program, the first data extraction sheet included the program name, program authors, country of origin, duration of program, frequency and intensity of training sessions, setting, program facilitators, age range for whom the program is intended, and data source(s) from which the program information was extracted. To gather data for the content analysis, the second data extraction sheet included: child skills (developmental areas targeted by the program), intervention details (intervention aim and philosophy), and caregiver intervention elements (overarching caregiver-coached strategies, indicators the coaching strategy is being enacted, and anticipated impact of the coaching strategy on the child).

### Content Analysis

Content analysis was selected as the method for identifying and comparing caregiver-mediated program elements extracted from the program materials. Content analysis is a systematic method that involves identifying the presence, meaning, and relationships across themes within qualitative research that can be used to build a conceptual model or system (Elo & Kyngäs, 2008). It is particularly useful for revealing patterns in qualitative content (Elo & Kyngäs, 2008; Vaismoradi et al., 2013) and enables the organization of large amounts of texts into categories. It has successfully been used in previously conducted studies to identify and categorize program elements and/or practices (see Frost et al., 2021; Pellecchia et al., 2024; Visscher et al., 2018). In this review, an inductive bottom-up approach was employed since it enabled the generation of codes/categories from the data directly, rather than being derived from a pre-existing framework or classification (Elo & Kyngäs, 2008).

Data were extracted independently by two coders and cross-reviewed after every three to four programs to identify any missing or inconsistent data entries. Discrepancies were resolved through discussions, with the senior author verifying each data entry with the resource material. Categories and codes were developed iteratively, and all program resources were revisited multiple times during data extraction and analysis to enhance accuracy and minimize human error. Given the substantial volume of textual data, and the iterative nature of the coding process, formal inter-rater reliability between coders was not calculated. Instead, analytic rigor was maintained through independent data extraction, cross-reviewing of data, regular and iterative consensus meetings (to refine codes, resolve any ambiguities while coding, and determine if additional codes were required to capture the intervention elements), senior author debriefing and verification, along with program developer confirmation of data to ensure validity. This approach enabled multiple perspectives within the research team to inform the development and refinement of codes and categories, thereby strengthening the robustness of the analysis. For those developers who responded with modifications, we requested written references to confirm their proposed modifications. Extracted data were validated by 13 of the 18 program developers or designated contact persons, following which suggested corrections or updates were made to the extracted data. See Figure 1 for more details on data extraction and validation.

**Figure 1.**
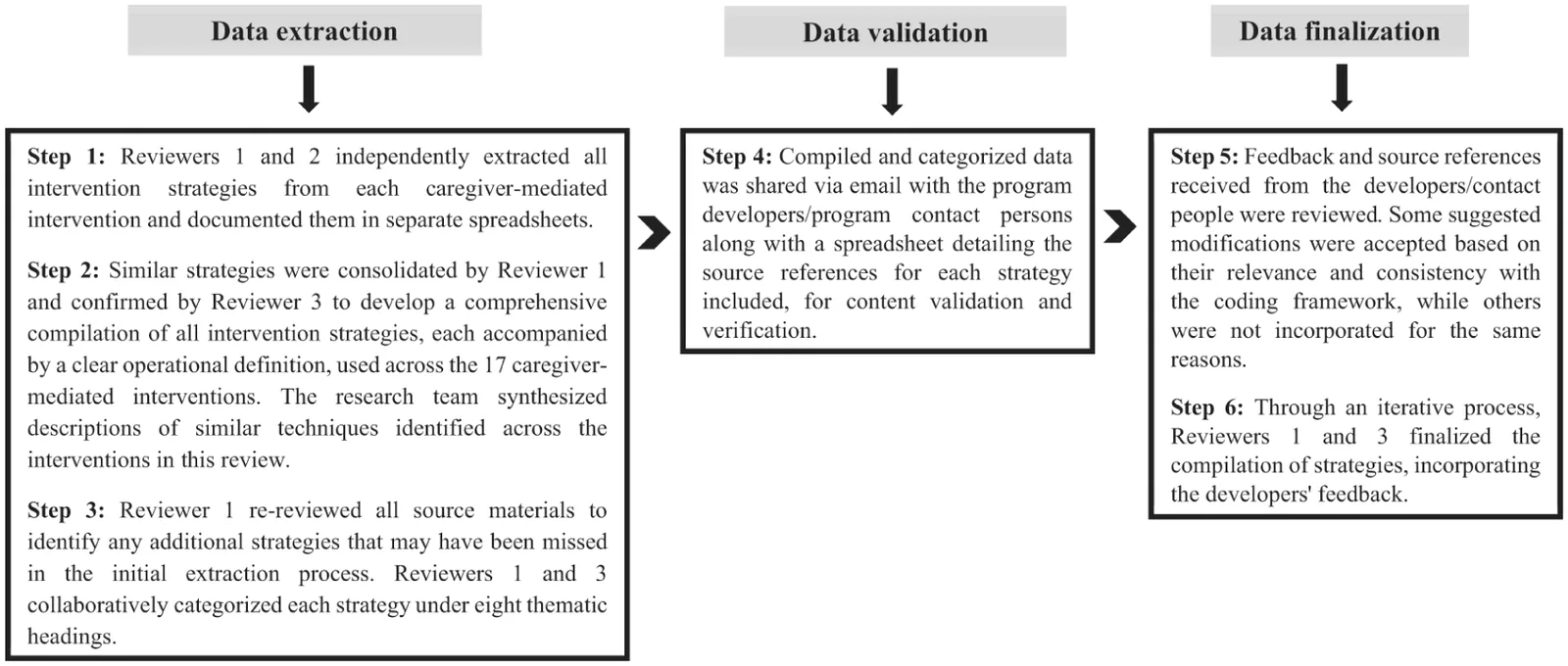
Process of extracting and validating intervention strategies.

## Results

Searches across seven databases yielded 2481 citations, of which 914 were screened, and 124 underwent full-text review (see Figure 2). Reviewers 1 and 2 completed title/abstract screening for 25% of the articles and achieved an inter-rater reliability of 97.8%. Reviewer 1 then independently screened the remaining 75% of the articles. Subsequently, both reviewers completed the full-text reviews. Cohen’s kappa was calculated based on the initial 25% of articles that were jointly screened by both reviewers. Cohen’s kappa was 0.57 for title/abstract screening and 0.55 for full-text review. The comparatively lower kappa value observed is due to the high prevalence of a single category in the subset (i.e., a higher proportion of articles being *excluded* rather than included in this case). This phenomenon, often referred to as the *Kappa Paradox*, occurs when high agreement coincides with a lower kappa statistic due to imbalanced category distribution (Bexkens et al., 2018; Zec et al., 2017). Ultimately, 67 studies met inclusion criteria, from which 21 caregiver-mediated, commercially available programs were identified.

**Figure 2.**
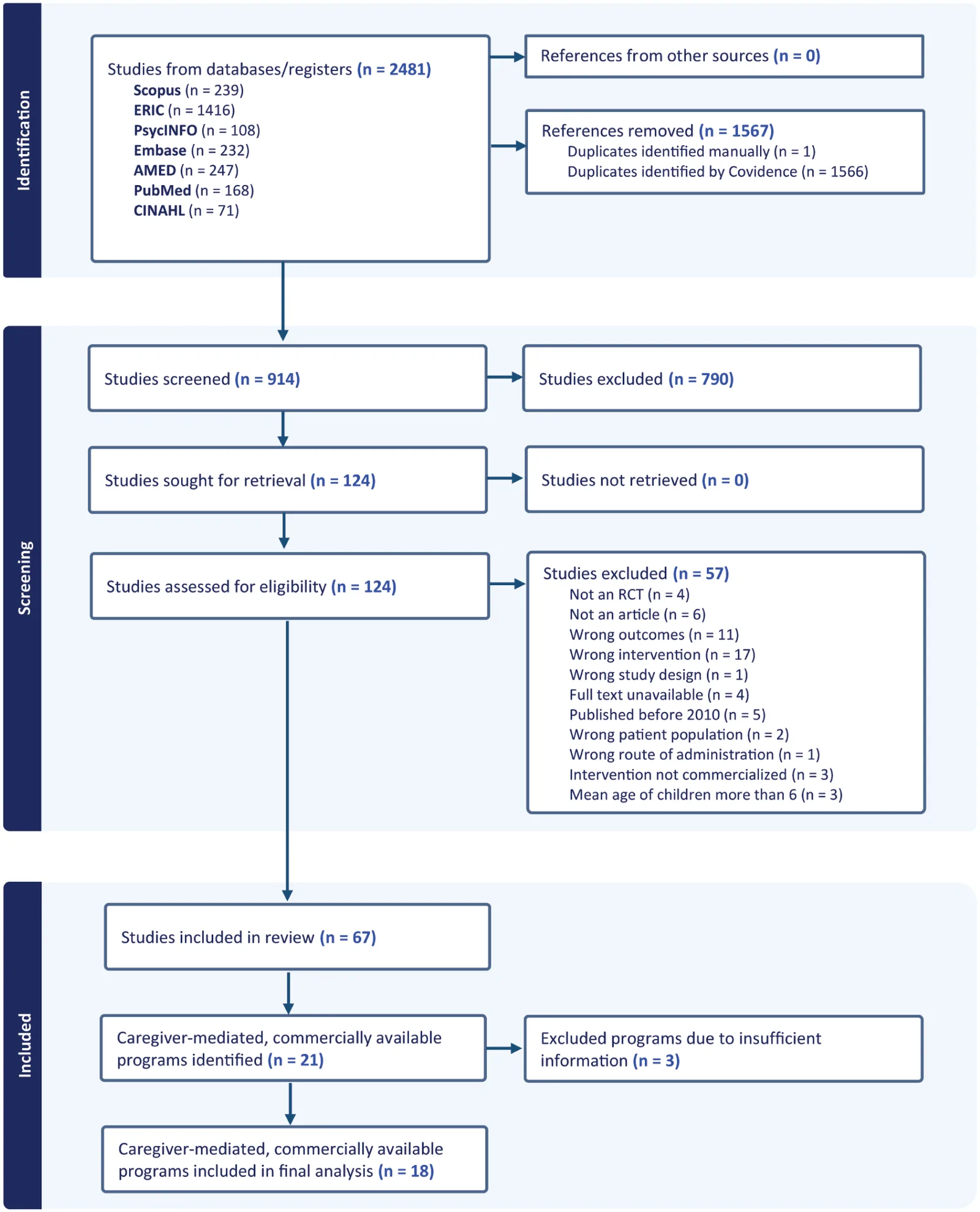
PRISMA diagram generated by Covidence.

Of the 21 programs, three were excluded (*Treatment and Education of Autistic and Communication Handicapped Children, Parent Education and Behavior Management*, and *Enhancing Interactions*) due to insufficient information to extract data. We received manuals, fidelity tools, articles, and/or parent training materials from the program developers/resource persons for *Developmental, Individual Difference, Relationship-based/Floortime* (DIR); *Inklings; Promoting First Relationships* (PFR); *PLAY Project; Enhanced Milieu Teaching* (EMT); *Focused Playtime Intervention* (FPI); *Pathways; Reciprocal Imitation Training* (RIT); *Project ImPACT*; and *Social ABCs.*

### Similarities and Differences in Child Skill Development Areas Targeted (Research Question 1)

To address our first research question, and to summarize intervention philosophy and basic program characteristics, we used publicly available information from 18 programs. See Supplemental Appendix D for resources used for data extraction.

Table 1 provides an overview of each of the included programs. Of the 18 programs, most were developed in North America, except for *Inklings* and *Paediatric Autism Communication Therapy* (PACT), both developed in England, and *World Health Organization–Caregiver Skills Training* (WHO-CST), developed in Switzerland. The programs vary in target age range (from infancy to adulthood) and are offered in multiple settings including in-person and virtually.

**Table 1.** Overview of Included Caregiver-Mediated Interventions.

Program	Authors	Country developed	Target age range	Program duration	Frequency and intensity of training sessions	Setting	Program facilitator
Developmental, Individual Difference, Relationship-Based)/ Floortime (DIR)	Greenspan & Wieder (2007)	USA	Birth through adulthood	Varies. **ICDL website:** (a) 12-week home program (b) Week-long intensive program **Literature:** 10 weeks to 1 year	Varies. **ICDL website:** specific frequency/intensity for 12-week home program not stated **Literature:** 1–4 × 2–5 hr sessions/week	In-person (center, home), telehealth	Registered professionals (e.g., SLP, OT, PT, social worker, educator) with DIR training (28 hr minimum)
More Than Words (MTW)	Sussman et al. (2024)	Canada	Children below 5 years	**Original program:** 13 weeks **Adapted program**: 8 weeks	1 session/week (1 orientation, 1 pre-program consultation, 8 group sessions, 3 individual video feedback), 2.5 hr per group session	In-person (home, clinic, any venue accommodating 8 families for group sessions), telehealth	Hanen-certified SLP/Ts
Inklings	Green et al. (2013); Whitehouse et al. (2021)	England	6–18 months	5–6 months	10 sessions (6 core and 4 focused), 60–75 min every 2 weeks	Home, research center, telehealth	Early childhood specialists, SLPs, psychologists, nurses, health students/therapy assistants. Practitioners from other backgrounds may be considered depending on qualifications and experience
Pediatric Autism Communication Therapy (PACT)	Aldred & Green (2009); Green et al. (2010)	England	2–11 years	6–12 months	Varies. **PACT website:** 12 × 1- to 1.5-hr sessions, once every 2 weeks, with optional monthly maintenance sessions **Literature:** 18 × 2-hr sessions every 2 weeks for 6 months, then 1 session/month for 6 months	Home, clinic, education context, telehealth	Certified professionals (psychologists, psychiatrists, SLPs, specialist teachers, therapists, audiologists, social workers, specialist nurses, health visitors) with Level 2 PACT training
PLAY Project	Solomon et al. (2007)	USA	14 months–6 years	12 months (in real-time practice, duration may extend for 2 years)	1- × 3-hr session/month or alternative schedules, such as 1 hr/week. Flexible model that can be adapted to fit agency’s structure.	Home, clinic, school, telehealth	Fully certified PLAY Project Consultants (SLPs, OTs, EI providers, early education teachers, social workers, counselors, psychologists, physicians, nurses) who have completed the 6-week course and 1 year of supervised training
Promoting First Relationships (PFR)	Kelly et al. (2008)	USA	0–5 years	10 weeks	1 hr/week	Home, clinic	Certified PFR Level 2 professionals (e.g., infant mental health specialists, child welfare providers, social workers, home visitors, early interventionists, family service workers, childcare providers, early childhood education teachers, public health nurses) who have undergone at least 15 weeks of training
Responsive Teaching (RT)	Mahoney et al. (2006)	USA	Below 6 years	Varies. **Literature**: ranges from 4–12 months	30- to 60-min session/week	Home, clinic, any setting that has sufficient floor space – library, school, community center, park/home or center	Certified RT providers (e.g., early intervention specialists including developmental specialists, teachers, parent educators, OTs, SLPs, nurses, psychologists, psychiatrists, social workers who received training from one of the developers of RT and guided feedback for 6 months
Social Communication, Emotional Regulation, Transactional Support (SCERTS)	Prizant et al. (2003)	USA	3–6.5 years Can be used with older individuals throughout the school years and beyond	**Literature:** from 5 to 10 months	45- to 60-min sessions, 2–3 times/week. Parents encouraged to deliver intervention in everyday activities for at least 25 hr.	Home, school, clinic community, vocational settings, small/large group setting, limited to less than 30 participants	Trained special education teachers, SLPs, OTs, PTs.
Early Start Denver Model (ESDM)	Rogers & Dawson (2010)	USA	12–60 months	Varies. **Literature:** from 10 to 26 weeks. Rogers et al. (2019) coached parents an additional 4 hr/month for 2 years.	**ESDM manual:** At least 20 hr/week for home-based sessions. In clinic-based sessions, typically involve parent and child attending for one to a few hours per week. **Literature**: from 0.5 to 2 hr/week.	Not tied to any one delivery setting. Can be delivered in home, clinic, center, inclusive preschools and telehealth	ESDM-certified early childhood professionals (e.g., special educators, OTs, SLPs, psychologists, ABA practitioners).
Enhanced Milieu Teaching (EMT)	Kaiser et al. (2000)	USA	2–7 years	Varies. **Literature:** 3–6 months	Varies. **Literature:** 1–3 × 20–60-min sessions/week	Home, clinic, school	EMT trained professionals who have achieved fidelity to intervention (e.g., special educators, SLPs)
Focused Playtime Intervention (FPI)	Siller et al. (2013)	USA	6 years and below	12 weeks	1 × 90-min session/week	Home	Trained FPI professionals (usually with background in developmental psychology/counseling) and at least 2 days (16 hr) of didactic training followed by hands-on training with at least 1 family
Joint Attention, Symbolic Play, Engagement and Regulation Intervention (JASPER)	Kasari et al. (2006)	USA	12 months to early childhood and older children	Varies. **Literature:** from 8 weeks to 3 months	1–3 × 30- to 60-min sessions/week (may vary based on child’s age and ability). However, the goal is for parents to use strategies throughout their day	Home, school, clinic, or other community setting, telehealth	JASPER trained and certified professionals (e.g., clinicians, educators, early interventionists, clinical psychologists, applied developmentalists, special educators, SLPs). Can also be implemented by trained and certified caregivers, paraprofessionals, other community members.
Pathways	Developed by Michelle Campbell & Renee Hoffman; described in Rollins et al. (2021)	USA	Birth to 5 years	Varies. **Literature:** from 12 to 15 weeks. In real-world practice, may be 2 years or more.	1.5 hr/week Intensity can be tailored to suit child’s need	Home	Certified Pathways trainers (e.g., early interventionists such as SLPs, early intervention specialists, OTs, PTs, mental health professionals)
Pivotal Response Training (PRT)	Koegel & Koegel (2019)	USA	Toddlers (0–2 years) to middle school-age (12–14 years)	Varies. **Literature:** from 10 to 24 weeks.	**Literature**: 60–90 min/week	Home, on-site PRT centers, telehealth	Level 5 Certified PRT professionals (MA- or PhD-level expert in PRT, psychologists, special education teachers, SLPs) or clinicians who demonstrated fidelity to intervention (under supervision of PRT trainer)
Project ImPACT	Ingersoll & Dvortcsak (2019)	USA	18 months to 6 years, although it has also been used with older children (up to about 12 years)	12 weeks	**Individual sessions:** 24 × 60- to 90-min sessions, 2/week, then monthly follow-ups **Group sessions:** 6 × 2-hr group sessions alternated with 6 × 1-hr individual sessions. Can be adapted based on child/caregiver needs	Home, clinic, classroom (for group sessions), Telehealth adaptation available	Certified professionals (early childhood professionals, SLPs, special educators, behavioral specialists, clinical psychologists, social workers, and OTs) who have received 6 months of training and demonstrated fidelity to intervention
Reciprocal Imitation Training (RIT)	Ingersoll & Schreibman (2006)	USA	20 months to 7 years. Can be carried out for adolescents	8–10 weeks	1–2 × 30–40-min sessions/week, may be preceded by a 90-min group training session	Clinic, research center, telehealth	RIT-certified therapists, trained undergraduate/graduate research assistants who have demonstrated fidelity
Social ABCs	Brian et al. (2016)	Canada	12–42 months	**Standard model:** 12 weeks **Group model:** 6 weeks	**Standard model:** (a) Weeks 1–8: 1-hr 1–1 home visits (with tapering frequency: 3 visits in week 1, 2 in week 2, and weekly visits from weeks 3–8); (b) Weeks 9 & 11: Phone call check-ins; (c) Weeks 10 & 12: 1 booster session/week. **Group model:** 1 × 1.5–2-hr group session/week + coaching sessions on tapering frequency: Weeks 1–3: 2 × 1-hr sessions/week, Weeks 4–6: 1 × 1.5-hr session/week	**Standard model:** home, community setting. **Group model:** clinic, center, telehealth	Professionals from a range of disciplines (e.g., psychologists, SLPs, behavior analysts, early child educators) who have (a) completed formal training with Social ABCs expert team (approximately 150 hr), (b) 3+ years of experience working with young autistic children and families.
WHO Caregiver Skills Training (CST)	WHO (2022)	Switzerland	2–9 years	From 6 to 12 weeks. Additional group sessions and home visits offered based on caregiver needs	9 × 2–2.5-hr group sessions, 3 × 1–1.5 individual sessions, 3 weekly or biweekly telephone calls	Research site, home, health facility, school, community-based facility, telehealth	Non-specialists (community health workers, social service workers, nurses, early childhood education facilitators, teachers, experienced caregivers) trained for a minimum of 8 days and gained experience from local master trainers and supervisors

*Note.* SLP: speech language pathologist, OT: occupational therapist, PT: physiotherapist, EI: early intervention, ABA: applied behavior analysis.

Eight programs were identified as a DSP intervention and 10 as NDBI. Notably, *Social Communication, Emotional Regulation, Transactional Support* (SCERTS) was identified in published literature as both NDBI (Schreibman et al., 2015) and DSP intervention (Binns & Oram Cardy, 2019). Given that the avoidance of explicit communication prompts is a core feature distinguishing DSP from NDBI programs (Binns & Oram Cardy, 2019), and since we did not find any evidence of directive communication prompts in the resource materials, we view SCERTS as best aligned with DSP models.

Three programs (i.e., Inklings, PACT, and RIT) focused specifically on social communication. The remaining 15 programs expanded beyond social communication and targeted a broader range of skills (e.g., play, sensory motor skills, cognition, regulation). Programs also varied in their aims (see Table 2). For instance, JASPER focused primarily on improving the child’s interaction with people and objects, while Inklings aims to help caregivers understand and adapt to their infant’s individual communication style to promote optimal social and communicative development.

**Table 2.** Aim, Philosophy, and Targeted Child Skill Development Areas in Included Caregiver-Mediated Interventions.

Intervention	Aim	Intervention philosophy	Child skill development area
DIR	To build holistic and healthy foundations for social, emotional, and intellectual capacities	DSP	1. Regulation 2. Shared attention/engagement 3. Reciprocity 4. Cognitive/problem-solving/ideation 5. Communication/language 6. Sensory motor capacities
MTW	To support the child’s social communication by integrating practical strategies into the caregivers’ daily interactions with their child including everyday routines	DSP	1. Back-and-forth social interactions 2. Turn-taking in routines 3. Understanding of language 4. Play exploration
Inklings	To help caregivers understand and adapt to their infant’s individual communication style to promote optimal social and communicative development	DSP	1. Language 2. Social skills
PACT	To guide caregivers in providing a timely, highly adapted interaction context (where communication responses and language match the child’s competence), enhancing communication and social interaction skills in neurodiverse and autistic children, while simultaneously nurturing a supportive interaction to foster child communication initiation and intentionality, reducing distress or non-functional responses and improving parent-child interactions, with a central focus on augmenting parental sensitivity and responsiveness through video-feedback methods	DSP	Social and communication skills related to reciprocal social, prelinguistic, pragmatic and linguistic development. 1. Shared attention 2. Communication initiation 3. Intentionality 4. Pragmatic communication
PLAY Project	To help parents develop a better connection with their child through play, and help the child improve their language, development, behavior, and social skills	DSP	1. Play 2. Social and communication development 3. Emotional development 4. Emotional regulation and shared attention
PFR	To enhance caregiver sensitivity and responsiveness to the child during interactions, fostering a deeper understanding of the child’s social and emotional needs through strength-based consultation strategies	DSP	1. Social and emotional skills 2. Behavioral and emotional regulation 3. Social attention 4. Play
RT	To help the caregiver maximize the potential of each of their routine interactions with their child so that they support and enhance the child’s development and well-being	DSP	1. Cognition 2. Communication 3. Social-emotional functioning
SCERTS	To directly address the core challenges of autism by focusing on building an individual’s capacity to initiate communication with a conventional symbolic system and to be actively engaged in emotionally satisfying relationships through effective reciprocal communication, emotional regulation, and transactional supports	DSP (Binns & Oram Cardy, 2019) and NDBI (Schreibman et al., 2015)	1. Social communication 2. Emotional regulation
ESDM	To increase the rates of development in all domains for autistic children (primary objective) and improve core domains associated with autism, such as social communication skills, that promote children’s ability to learn from everyday experiences and interactions	NDBI	1. Receptive and expressive communication* 2. Imitation* 3. Social skills* 4. Play skills* 5. Joint attention 6. Cognitive skills 7. Fine motor skills 8. Gross motor skills 9. Self-care skills * *Given more importance*
EMT	To use children’s interests and initiations as opportunities to model and prompt language in everyday contexts	NDBI	1. Engagement 2. Language and communication 3. Play
FPI	To increase responsive parental behaviors and child communication in the context of collaborative toy play between the parent and child	NDBI	1. Coordinated attention and engagement 2. Play 3. Expressive language 4. Communication 5. Attachment behavior
JASPER	To improve children’s relationships with people (through joint attention and engagement) and interactions with objects (through development of play), while helping children maintain and modulate their affect and behavior (through regulation strategies)	NDBI	Facilitate growth in social communication skills across four domains: 1. Joint attention 2. Simple to symbolic play 3. Engagement 4. Regulation
Pathways	To collaboratively partner with IDEA Part C Programs, providing affordable and evidence-based early intervention for toddlers suspected of or diagnosed with autism, with a specific focus on developing foundational social communication skills, and empower parents to address their toddler’s unique needs and alter their developmental trajectory to foster active participation in the social environment, thereby minimizing the effects of autism	NDBI	1. Play 2. Imitation 3. Early foundational social communication skills (including eye contact, social engagement and verbal reciprocity) 4. Adaptive functioning 5. Behavioral regulation
PRT	To move autistic individuals toward a typical trajectory by targeting a broad spectrum of behaviors, fostering widespread collateral improvements in communication, socialization, and behavior within natural, inclusive settings, while empowering family members to seamlessly integrate evidence-based interventions into daily routines	NDBI	1. Motivation 2. Responsivity to multiple cues 3. Self-management 4. Social initiations 5. Speech and language skills 6. Play
Project ImPACT	To increase the parent’s responsiveness to the child’s behavior and teach the child to use new communication, imitation, and play skills within ongoing interactions in daily routines	NDBI	1. Social engagement 2. Communication 3. Imitation 4. Play
RIT	To establish back-and-forth social games/interaction in which the parent and child imitate each other	NDBI	1. Imitation 2. Social engagement 3. Joint attention 4. Gestural usage
Social ABCs	To foster vocal social communication that is intentional, directed, and functional, and encourage positive emotion sharing between the child and the caregiver	NDBI	1. Communication (intentional, directed, functional vocal communication) * 2. Positive emotion sharing (i.e., sharing meaningful, positive emotions with a primary caregiver) * 3. Emotional regulation 4. Play *Given more importance
WHO-CST	To provide caregivers with skills they can use at home to improve their child’s engagement in activities and communication and promote positive behavior and skills for daily living.	NDBI	1. Communication 2. Shared engagement 3. Reducing challenging behaviors 4. Problem-solving

### Similarities and Differences in Caregiver-Coached Strategies (Research Question 2)

For our second research question, access to manuals, fidelity tools, and other program resources was required. We were able to obtain this information for 17 of the 18 programs. We were unable to access sufficient materials for *Pivotal Response Training* (PRT), and therefore it was not included in the analyses addressing the second research question. A total of 70 unique caregiver-coached strategies were identified across 17 programs (see Table 3 and Tricco et al., 2018), as detailed in supplementary spreadsheet). These were initially extracted from fidelity tools; however, we did not have access to all fidelity tools, and those available were not always comprehensive. Therefore, a thorough review of program manuals was conducted to generate a comprehensive list of caregiver-coached strategies. These were then thematically grouped into eight categories based on the developmental skills they support. These included strategies that

1. support prelinguistic skills and engagement (*n* = 10)
2. facilitate learning of new skills (*n* = 9)
3. support expressive communication and comprehension (*n* = 18)
4. support play development (*n* = 6)
5. support engagement through environmental arrangement (*n* = 4)
6. support emotional development through interpersonal/relational supports (*n* = 7)
7. prevent challenging or interfering behavior (*n* = 3)
8. support the child in times of distress or when expressing sensory needs (*n* = 13).

**Table 3.** Intervention Strategies in Included Caregiver-Mediated Interventions Grouped Thematically by Child Development Areas Supported.

Intervention strategies	DSP								NDBI								
	DIR	MTW	Inklings	PACT	RT	PFR	PLAY Project	SCERTS	ESDM	Project ImPACT	RIT	Social ABCs	Pathways	FPI	EMT	JASPER	WHO-CST
**To support prelinguistic skills and engagement**																	
Build and sustain reciprocal back-forth interaction (e.g., through rhythm, facial expressions, gestures, scaffolding)	✓	✓	✓	?^D^	✓	?^D^	✓	-?	✓	✓	✓	-	✓	-	-?	✓?	-?
Be sensitive and responsive to child’s communication cues (e.g., look, respond to spoken & non-spoken communication)	✓	✓	✓	✓	✓	✓	✓	✓?	✓	✓	-	✓	✓	✓	✓?	✓?	✓?
Follow the child’s lead	✓	✓	✓	✓	✓	✓	✓	✓?	✓	✓	✓	✓	✓	✓	✓?	✓?	✓?
Find the balance between following and leading the interaction	-	?^R^	-	-	-	-	✓	✓?	✓	✓	✓	✓	✓	✓	-?	✓?	-?
Use and adjust affect/animation to help child stay regulated, aroused, attentive and motivated	✓	✓	✓	?^D^	✓	✓	✓	✓?	✓	✓	✓	✓	✓	✓	-?	✓?	-?
Observe, accept and adapt to the child’s unique style of engaging (e.g., not pressuring the child to make eye contact)	✓	✓	✓	?^D^	-	✓	✓	-?	?^D^	-	-	?^D^	?^D^	-	-?	-?	-?
Imitate the child	✓	✓	✓	?^D^	✓	✓	✓	✓?	✓	✓	✓	-	✓	✓	✓?	✓?	✓?
Stay face-face with the child/get down to child’s level	✓	✓	✓	-	✓	✓	✓	✓?	✓	✓	✓	✓	✓	-	✓?	✓?	✓?
Engage in turn taking (e.g., use anticipatory phrases to warn the child, make adult’s turns short at first, use adult’s turn to model new ways to play with the object)	✓	✓	-	?^D^	✓	-	✓	✓?	✓	✓	-	-	✓	✓	✓?	✓?	-?
Encourage eye contact to mand for objects (e.g., get into the child’s line of sight through prompts/reinforcement/waiting/blocking)	-	-	-	-	-	-	-	-?	✓	-	-	-	✓	?^D^	-?	-?	-?
**To facilitate learning of new skills**																	
Provide behavioral teaching trials (e.g., ABC)	-	-	-	-	-	-	-	-?	✓	✓	✓	✓	✓	-	✓?	✓?	-?
Provide instructional teaching techniques (e.g., prompting, fading, shaping, chaining) to teach communication, play, etc.	-	-	-	-	-	-	-	✓?	✓	✓	✓	✓	✓	✓	✓?	✓?	-?
Provide extrinsic reinforcement (e.g., social reinforcers, edible reinforcers, etc.) any time the child’s response matches the caregiver’s expectation (for a “good” response)	-	?^R^	-	-	-	-	✓	✓?	?^R^	✓	✓	-	✓	✓	✓?	-?	✓?
Provide natural reinforcement (e.g., desired object given to child after the child requests for it) any time the child’s response matches the caregiver’s expectation (for a “good” response)	-	✓	-	-	-	-	?^D^	-?	✓	✓	✓	✓	✓	-	✓?	✓?	✓?
Provide reinforcement for all attempts made by the child irrespective of whether child’s response matches caregiver’s expectation	-	✓	-	-	-	✓	-	-?	?^D^	✓	-	?^D^	-	-	-?	-?	✓?
Establish and/or elaborate joint activity structure (beginning-middle-end)/routines	?^D^	✓	✓	✓	✓	✓	✓	✓?	✓	✓	✓	✓	✓	✓	✓?	✓?	✓?
Use time delay strategies (e.g., wait for child to react after providing an opportunity to respond, take one turn and wait)	✓	✓	✓	✓	✓	-	✓	✓?	✓	✓	✓	✓	✓	✓	✓?	✓?	✓?
Use cognitive scaffolding (e.g., offering hints, visuals; playful obstruction, using gestures, reducing barriers to success, structuring the environment to ensure the child can meaningfully explore)	✓	✓	-	-	✓	✓	✓	✓?	✓	✓	-	-	✓	-	-?	✓?	✓?
Provide hand-over-hand guidance/gentle physical guidance when the child is unable to provide a correct/target response	-	?^R^	-	-	-	-	-	-?	?^D^	✓	✓	-	✓	-	-?	✓?	✓?
**To support expressive communication and comprehension**																	
Talk about what the child is seeing, hearing or doing (i.e., commenting)	✓	✓	✓	✓	✓	✓	✓	✓?	✓	✓	✓	-	✓	✓	✓?	✓?	✓?
Avoid asking a lot of questions or making directive commands	✓	✓	✓	✓	✓	-	✓	✓?	✓	✓	✓	✓	✓	✓	✓?	✓?	✓?
Model language that is within the child’s perceived zone of proximal development	✓	✓	-	✓	✓	-	✓	✓?	✓	✓	✓	-	✓	✓	✓?	✓?	✓?
Say and repeat familiar words/phrases while interacting with the child (i.e., modeling, focused stimulation)	-	✓	-	✓	-	-	✓	-?	✓	✓	✓	-	✓	-	-?	-?	-?
Provide multiple and varied communication opportunities in an activity	?^D^	✓	✓	✓	-	?^D^	✓	✓	✓	✓	-	✓	?^D^	-	-?	-?	-?
Speak slowly/emphasize target words while interacting with the child	-	✓	-	?^D^	-	-	✓	-?	-	✓	✓	-	✓	✓	-?	-?	-?
Ensure child is interested and paying attention then connect what the child sees/wants with hearing its label, trying to say it, and getting it	-	-	-	-	-	-	?^D^	-?	-	?^D^	-	✓	-	-	-?	-?	-?
Clearly point to or show objects while verbally labeling them to make the object names more salient.	-	✓	-	-	✓	-	✓	*-*?	✓	-	-	-	-	-	✓?	✓?	-?
Balance the adult’s use of communicative functions	-	?^D^	-	-	-	-	-	✓?	✓	-	-	-	-	-	-?	-?	-?
Provide comments that are contextual, simple, and do not contain words that are the target goals for the child (i.e., model prompts)	-	-	-	-	-	-	-	-?	-	-	-	✓	-	-	-?	-?	-?
Use contextual/environmental cues to amplify meaning of targeted aspects of language	✓	?^D^	-	-	-	-	-	-?	?^D^	✓	-	-	-	-	✓?	-?	-?
Provide explicit prompts for communication (e.g., prompt target word/action)	-	?^R^	-	-	-	-	-	-?	✓	✓	✓	✓	✓	✓	✓?	✓?	-?
Use communicative scaffolding to support responding (e.g., provide choices, make deliberate mistakes for the child to correct)	✓	?^R^	-	✓	✓	✓	✓	✓?	✓	✓	-	-	✓	✓	✓?	✓?	✓?
Entice the child to initiate (e.g., by creating opportunities for communication; communicative temptations)	✓	?^R^	-	✓	-	✓	✓	✓?	✓	✓	-	✓	✓	✓	✓?	✓?	✓?
Respond to the child’s intention even if the child’s word choices/pronunciations may not be the expected response	✓	✓	✓	-	✓	✓	✓	✓?	-	✓	-	?^D^	?^D^	-	-?	✓?	✓?
Restate/recast the child’s utterance	✓	✓	-	✓	✓	✓	✓	✓?	✓	✓	✓	✓	✓	?^D^	-?	✓?	✓?
Expand the child’s utterance	?^D^	-	-	✓	✓	-	✓	✓?	✓	✓	✓	✓	✓	-	✓?	✓?	✓?
Utilize AAC to make it easier for the child to interact	✓	?^R^	-	-	-	-	-	✓?	✓	-	-	-	✓	-	✓?	✓?	-?
**To support play development**																	
Model play to the child based on the child’s functional developmental level	-	?^R^	-	-	✓	✓	✓	✓?	✓	✓	✓	✓	-	✓	✓?	✓?	✓?
Provide multiple and varied play opportunities in an activity	✓	-	-	?^D^	-	✓	✓	-?	✓	✓	✓	✓	-	-	-?	✓?	✓?
Use toys creatively (depart from conventional ways of using the toys and adapt to the child’s way of playing)	✓	?^D^	✓	?^D^	-	✓	✓	-?	?^D^	-	✓	✓	?^D^	✓	✓?	-?	-?
Let child initiate the play idea and through interaction (dramatization in roles), elaborate with expanding themes and range of emotions that the child can explore and express safely	✓	?^D^	-	-	-	?^D^	✓	-?	✓	-	-	-	-	-	-?	-?	-?
Expand the child’s play interests/play	✓	✓	✓	✓	✓	✓	✓	✓?	✓	✓	-	✓	✓	✓	✓?	✓?	✓?
Model appropriate/conventional play when the child uses inappropriate/unconventional play	-	?^R^	-	-	-	-	-	✓?	-	✓	-	✓	✓	-	-?	✓?	-?
**To support engagement through environmental arrangement**																	
Create clearly defined spaces & materials and limit distractions and clutter	✓	-	-	?^D^	✓	✓	✓	✓?	✓	✓	✓	-	✓	✓	✓?	✓?	✓?
Support transition between activities (e.g., provide warnings)	-	-	-	-	-	✓	-	✓?	✓	✓	-	✓	?^D^	✓	✓?	✓?	✓?
Provide assistive visual or organizational supports (when appropriate)	✓	?^R^	-	-	-	-	✓	✓?	✓	-	-	-	-	-	✓?	✓?	✓?
Use sensorimotor and/or motor supports	✓	?^R^	✓	-	-	✓	✓	✓?	✓	-	-	-	-	-	-?	-?	-?
**To support emotional development through interpersonal/relational supports**																	
Be physically available and interactive	✓	?^D^	✓	?^D^	✓	✓	✓	-?	✓	-	-	✓	-	-	-?	-?	-?
Use a range of affective cues and responses (e.g., intonation, facial expressions, gestures, non-verbal communication)	✓	✓	✓	?^D^	✓	✓	✓	✓?	✓	✓	-	-	✓	-	-?	✓?	-?
Use positive affect/positive emotion sharing	-	-	-	?^D^	✓	✓	✓	-?	✓	✓	-	✓	✓	✓	-?	✓?	✓?
Accept, empathize, and validate all the child’s feelings, even negative ones	✓	?^D^	✓	?^D^	✓	✓	✓	-?	✓	-	-	?^D^	-	-	-?	-?	-?
Recognize and label the child’s feelings	-	✓	✓	?^D^	✓	✓	✓	-?	✓	✓	-	-	-	-	-?	-?	-?
Use touch, voice, body language and facial expressions to help child understand their feelings	-	✓	✓	-	✓	✓	✓	-?	✓	-	-	-	-	-	-?	-?	-?
Teach child acceptable ways of expressing strong feelings (e.g., provide concrete suggestions for what the child can say or do to express their strong feeling)	-	?^R^	-	-	✓	✓	?^D^	-?	-	-	-	-	-	-	-?	-?	-?
**To prevent challenging or interfering behavior**																	
Identify trigger(s)/antecedent(s) that may underlie the child’s interfering behaviors and employ strategies to alleviate the distress	-	-	✓	-	-	✓	-	-?	✓	✓	-	✓	✓	✓	-?	✓?	✓?
Give clear instructions and set expectations to prevent challenging behaviors	-	-	-	-	✓	✓	-	-?	?^D^	✓	-	-	-	-	✓?	✓?	✓?
Catch the child exhibiting ‘good’ behavior and praise them for it	-	-	-	-	-	✓	✓	-?	-	-	-	-	✓	-	✓?	✓?	✓?
**To support child in times of distress or when expressing sensory needs**																	
Redirect away from interfering behaviors	✓	-	✓	-	✓	✓	-	-?	✓	✓	-	✓	✓	✓	✓?	✓?	-?
Ignore the ‘challenging’ behavior	-	-	-	-	✓	-	✓	-?	-	-	-	✓	✓	-	✓	✓	✓?
Pause and take a few breaths, use self-talk, and repeat a key word that calms the caregiver during moments of disruption in parent–child relationship	-	-	-	-	-	✓	-	-?	-	-	-	-	-	-	-?	-?	-?
Respond sensitively to the child’s distress by using gentle touch, tone of voice, facial expressions, rocking, holding, singing, and/or offering comfort to co-regulate the child	✓	?^D^	✓	?^D^	✓	✓	✓	*-*?	-	-	-	✓	-	-	-?	-?	-?
Facilitate experiences for the child that satisfy sensory needs akin to their repetitive/self-stimulatory behavior	✓	✓	✓	-	-	-	✓	-?	-	-	-	?^D^	✓	-	-?	-?	✓?
When the child is distressed/ dysregulated, change the activity being done, lessen the stimulation, or take a break	✓	?^D^	-	-	-	✓	-	✓?	✓	?^D^	-	✓	-	✓	-?	✓?	-?
Encourage self-soothing (e.g., offer blankets, stuffed animal, something to suck on)	-	-	-	-	-	✓	-	-?	-	-	-	✓	-	-	-?	-?	-?
Use language to re-interpret and describe the child’s actions in a way that assumes positive intention (e.g., if the child throws a toy car in the corner, the parent may say “all done car”)	-	-	✓	?^D^	-	-	✓	✓?	-	-	-	-	-	✓	-?	-?	-?
Respond to challenging behavior that aims to get access by teaching the child to use communication skills	-	-	-	-	-	-	✓	-?	?^D^	✓	-	-	-	-	-?	✓?	✓?
Model appropriate/conventional behavior when the child uses inappropriate/unconventional behavior if the child is regulated enough to benefit from models	-	-	-	-	-	-	-	✓?	✓	✓	✓	-	-	-	-?	✓?	✓?
Use reinforcement strategies to teach adaptive/conventional behaviors (e.g., positive behavior supports)	-	-	-	-	-	-	-	-?	✓	✓	-	-	✓	-	-?	-?	-?
Provide discipline with choices/limits/responsive guidance	✓	-	-	-	✓	✓	✓	-?	-	✓	-	-	-	-	-?	-?	-?
Provide a 3-step follow-through when the child exhibits an inappropriate behavior	-	-	-	-	-	-	-	-?	-	-	-	-	-	-	✓?	-?	-?

**Table legend** ✓ Caregivers are coached to use strategy—coded by reviewers and confirmed by developers - Caregivers are **NOT** coached to use strategy—coded by reviewers and confirmed by developers ?^D^ Developers coded strategy as coached, but reviewers did not–disagreement or lack of access to resources ?^R^ Reviewers coded strategy as coached, but developers did not—lack of access to updated versions of resources ✓? Caregiver are coached to use strategy according to reviewers only - developers did not respond -? Caregivers are NOT coached to use strategy according to reviewers only–developers did not respond

Category 1 strategies that support prelinguistic skills and engagement such as “Be sensitive and responsive to the child’s communication cues,” and “use and adjust affect/animation” were reported in the majority of programs. Other strategies common across all 17 programs included following the child’s lead and commenting. Most programs also employed strategies for establishing joint activity routines (*n* = 16), using time delays (*n* = 16), avoiding asking a lot of questions (*n* = 16), expanding play (*n* = 16), imitating the child (*n* = 15), and staying face-to-face with the child (*n* = 15). Some strategies unique to only a few programs included “providing comments that are contextual, simple, and do not contain words that are target goals for the child” (Social ABCs), teaching the child acceptable ways of expressing strong feelings (RT, PFR), “providing a 3-step follow through when the child exhibits an inappropriate behavior” (EMT), “ensuring the child is interested and paying attention, then connecting what the child sees/wants with hearing its label, trying to say it and getting it” (Social ABCs), and encouraging eye contact to mand for objects (ESDM, Pathways).

Once the extracted data were verified by 13 of the 18 program developers or designated contact persons, the study team agreed to revise the data in alignment with the developers’ proposed changes when supporting evidence was provided. However, changes were not made in some instances due to lack of access to written evidence (e.g., no access to the updated version of the MTW program, or the PACT manual to validate proposed edits). We did not receive responses to our requests to verify the extracted data from the developers of four programs (SCERTS, JASPER, WHO-CST, and EMT). The coding of intervention strategies in Table 3 includes representative agreement (or the lack thereof) for each program and strategy. Agreement between the study team and the developers who responded to the intervention strategies extracted ranged from 71% to 100% (see Supplemental Appendix E). Supplemental Appendix F contains the references for the specific strategies mentioned in each of the programs.

## Discussion

The number of commercially available programs supporting social communication for autistic children has grown over the years. While some programs share similarities in their theoretical foundation and elements, they also present distinct features. To support the field’s shift toward precision and individualized care (Gabis et al., 2021), we examined the similarities and differences in the developmental domains targeted and program philosophies across 18 identified social communication programs, as well as the specific strategies coached to caregivers in 17 of these programs. These 18 programs were relatively balanced in their alignment with either DSP (*n* = 8) or NDBI (*n* = 10) program models.

### Research Question 1

Our first research question sought to identify similarities and differences in specific child skill development areas supported within the programs. Of the 18 programs included, three focused exclusively on social communication, while 15 supported a broad range of developmental domains in addition to social communication. Identifying differences across programs can support clinicians and families to make informed choices regarding which program might align best with their child’s developmental profile. From a research perspective, these findings can aid researchers in developing more nuanced and aligned research questions and designing future comparative effectiveness studies. For example, programs identified as being more holistic would be well-suited for studies designed to explore the synergetic effects of supporting multiple developmental areas simultaneously or sequentially.

### Research Question 2

Our second research question aimed to identify similarities and differences in the overarching strategies that caregivers were coached to adopt. Across the 17 programs, 70 strategies were identified, including many shared and some unique program elements. Commonly shared strategies included following the child’s lead, being sensitive and responsive to the child’s communication cues, imitating the child, establishing joint activity routines, expanding the child’s play, avoiding asking a lot of questions and/or making directive commands, and commenting, among others. Examples of unique techniques specific to only a few programs included encouraging eye contact, teaching the child *acceptable* ways of expressing strong feelings, providing a three-step follow-through when the child exhibits inappropriate behavior, balancing the caregivers’ use of communicative functions, and engaging the child in self-soothing, among others.

Despite common strategies identified across the 17 caregiver-mediated programs, we found variations in how programs implemented the same strategy. This can be problematic for researchers conducting systematic reviews and meta-analyses where there is an assumption of some level of intervention similarity. If strategies vary in their implementation, combining results becomes problematic. This was evident particularly for the strategies of commenting, following the child’s lead, and providing behavioral teaching trials to the child. For example, many programs encouraged commenting; however, some programs encouraged specific, nuanced ways of commenting. For example, Social ABCs encourages providing comments that are contextual, simple, and do not contain the words that are target goals for the child (i.e., “model prompts”). In contrast, other programs encourage commenting more broadly, such as talking about what the child is seeing, hearing, or doing (e.g., MTW, ESDM, Pathways, PLAY Project, among others). This example exemplifies meaningful variation in how caregivers are being coached to use comments to support children’s social communication and highlights the importance of specificity in describing caregiver-coaching strategies.

Following the child’s lead was also a caregiver-coaching strategy commonly identified across all 17 programs. Although this shared terminology consistently appeared in program manuals and/or fidelity tools, the stated enactment of this strategy differed across programs. For example, RT encouraged caregivers to engage in activities in the same manner as the child and respond to the child’s interests. Similarly, the DIR fidelity measure noted attending to the specific aspect of their child’s focus of attention and joining them in interaction through this shared focus/interest. Project ImPACT provided detailed guidance, including being face-to-face, joining in on the child’s play, avoiding questions and directives, being sensitive but persistent, setting limits, and waiting and responding to all the child’s actions. Similarly, MTW guided caregivers to notice and join the child’s actions, interpret behaviors as communicative, and imitate the child’s sounds and movements. JASPER recommended following the child’s lead and choices as much as possible unless the play was repetitive or “unproductive.” Social ABCs suggested following the child’s lead but supporting transitions to more functional activities whenever required. In contrast, PFR emphasized observing the child’s interests, engaging at the child’s level, imitating, and allowing exploration without imposing conventional play expectations. Similarly, in FPI, caregivers are coached to respond to their child’s interests and preferences. However, when children demonstrate repetitive play, caregivers are guided to adopt one of two approaches: either continue to follow the child’s lead or imitate the repetitive actions, even if it is unconventional, to establish shared attention; or redirect the child’s attention to another activity, emphasizing a flexible approach to following the child’s lead, based on the caregiver’s preference.

Similarly, there were nuances in the way that programs using behavioral teaching trials described their implementation. For example, in ESDM, caregivers are encouraged to provide behavioral teaching using the ABC format, with frequent and repetitive teaching trials during play every 30 s, whereas in RIT, caregivers are encouraged to implement the RIT teaching cycle six to eight times within a 20-min session. These variations in *how* caregivers are coached to deliver teaching trials to their child underscore the importance of understanding program-specific approaches to ensure alignment with the child’s developmental profile and the family’s preferences.

The aforenoted examples exemplify semantic alignment without operational equivalence. This may create an impression of coherence within the evidence base while obscuring meaningful variation in fidelity and potential mechanisms of change. This could ultimately limit the ability to capture potentially meaningful differences across programs and/or participants. For clinicians and policy makers who rely on evidence to choose intervention programs, it may lead to confusion and inconsistent translation into practice.

Notably, some of the differences in caregiver-coached strategies across the programs align with movement toward more neuro-affirming practices, while others do not. Dallman et al. (2022) identified eight principles of neuro-affirming practice, including: (a) accepting the client’s unique style of neuroprocessing, (b) consulting with neurodivergent individuals, (c) avoiding descriptors such as desirable/undesirable behaviors or labeling clients as complaint/non-compliant, (d) prioritizing intervention goals that are based on the client’s interest and emotional well-being, (e) enabling the right to stim, (f) guaranteeing the right to socially engage in ways that they are comfortable, (g) honoring all forms of communication, and (h) practicing reflexivity. In line with these tenets of neuro-affirming practice, this review identified few strategies that align with neuro-affirmative principles (even though they were not explicitly labeled as such by program developers) such as following the child’s lead, using toys creatively (i.e., adapting to the child’s way of playing, even if it’s unconventional), accepting and adapting to the child’s unique style of engaging, and facilitating experiences for the child that satisfy sensory needs akin to their repetitive/self-stimulatory behavior. However, the extent to which these strategies were conceptualized and implemented might have varied across programs and warrants for further examination. In addition, incorporating perspectives from individuals with lived experiences would be valuable in assessing the degree to which these strategies are in fact neuro-affirming.

Encouraging eye contact is one practice not viewed as neurodiversity-affirming (Moore et al., 2024). Two of the 17 programs included in this review (Pathways and ESDM) explicitly mentioned coaching caregivers to prompt their child to make eye contact to mand for objects. Notably, Rollins (2017) reported that a key differentiating feature of Pathways was the “facilitation of social eye contact without aversive prompts” (p. 15). The WHO-CST manual identified eye contact as a goal for children who have not started requesting, but the way in which caregivers were instructed to use this strategy was not clearly described. A few other programs did not explicitly mention eye contact as a targeted child skill development area but included it in descriptions of shared attention and social engagement, with some of these programs using strategies that could naturally result in the child using eye contact. These strategies included sitting face-to-face with the child at a reasonably close distance and smiling at the child. Nonetheless, the low number of programs that explicitly target eye contact may reflect a broader shift in the field away from earlier, compliance-focused models of autism interventions and more toward more responsive and neurodiversity-affirming practices.

Another noteworthy observation was different patterns of strategies coached based on the theoretical underpinnings (DSP or NDBI) of the programs. For example, most DSP programs implemented the strategy of observing, adapting to, and accepting the child’s unique style of engaging, responding sensitively to the child’s distress, and providing a range of sensorimotor, interpersonal, and relational supports. One strategy unique to most NDBIs incorporated coaching caregivers to use behavioral teaching trials, instructional teaching techniques, explicit prompting for communication, and extrinsic reinforcement strategies, thus reflecting the integration of applied behavior analysis (ABA) principles within a developmental framework.

Although many caregiver-mediated interventions use program-specific fidelity tools that monitor changes in caregiver’s use of strategies over time, emerging cross-program fidelity measures may offer a more standardized approach. For example, the Measure of NDBI Strategy Implementation – Caregiver Change (MONSI-CC; Vibert et al., 2020) and NDBI-Fi (Frost et al., 2020) show promise in capturing caregivers’ implementation of NDBI strategies during interactions with their child. The use of such measures could facilitate comparisons of caregiver implementation across interventions that are grounded in the NDBI philosophy.

However, during data extraction, we observed mismatches in the strategies mentioned in the program manuals and those listed in the fidelity tools; that is, reviewers were often able to find strategies listed in the fidelity measures within the manuals, but not vice versa. This suggests that complete reliance on only fidelity tools in their current form may not provide a valid measure of caregiver fidelity. Fidelity tools are a critical component of caregiver-mediated interventions as they enable evaluation of caregivers’ implementation of strategies. Ensuring that program fidelity tools capture all targeted strategies is essential to evaluating program effectiveness and caregiver adherence.

All of the common and essential elements of NDBIs identified by Frost et al. (2020) were also captured in the current review. These included being face-to-face and on the child’s level, following the child’s lead, modeling language, positive affect and animation, responding to the child’s attempts to communicate, using communicative temptations, and the frequency and quality of direct teaching episodes. Cullinane et al. (2024) identified the common strategies employed in Developmental Relationship-Based Interventions including social play, sensitive responding, following the child’s lead, and presenting challenges, all of which were reflected in caregiver-mediated programs included in the current review. What distinguishes the current review is not merely the confirmation of previously identified elements across programs, but its broader comparative scope as we synthesized evidence across a wide range of caregiver-mediated programs, unlike previous studies that focused on a single class of interventions. This review showcases a more fine-grained identification of practices, revealing many additional shared and unique caregiver-coached strategies that were not previously documented. Further, it identified semantic alignment across strategies without functional equivalence in enactment. Importantly, our analysis moves beyond cataloging strategies within the context of program manuals and fidelity tools, revealing gaps and mismatches that have implications for how program elements are defined, operationalized, and ultimately tested as mediators and moderators of effectiveness.

### Strengths and Limitations

The findings of this review have several important clinical and research implications. From a knowledge translation perspective, this review empowers clinicians and caregivers with a better understanding of the available interventions and can support informed decision-making. There is a need to develop more precise, operationalized fidelity measures that clearly define how a specific strategy, such as “follow your child’s lead,” will be implemented in practice. This will ensure accurate assessment of caregiver adherence to strategies and support cross-program comparisons. Future research should examine how specific caregiver-coached strategies contribute to various developmental outcomes, such as social communication and play. Equipped with the individual strategies identified in this review, the next logical step would be to determine the active ingredients that bring about a change in individual child outcomes and differentiate them from less or non-essential components. This can be achieved by designing experimental trials that adjust intervention strategies and evaluating the outcomes to identify casual relationships, if any, between the active ingredients and the outcomes (Frost et al., 2021). Identification of the key levers of change may also inform the development of novel or integrated interventions by systematically combining active ingredients across programs targeting similar goals (Abry et al., 2015).

It is important to acknowledge that, in some programs, intervention strategies may be coached to caregivers in a structured and cumulative manner. Therefore, the child’s responses may not be attributable to a new individual strategy alone but may also be influenced by previously learned strategies. This overlap may pose a challenge when trying to understand the specific effects of an individual strategy. At the same time, it also reinforces the idea that these caregiver-coached intervention strategies are designed to be used in an integrated manner (Frost et al., 2021). By outlining the individual strategies across programs, this review provides a foundation for examining how specific strategies cluster and are implemented in combination, thereby enabling a clearer understanding of patterns of strategy use and their potential combined effects.

While this review identified similarities and differences across commercialized programs, we recognize that in real-world practice, the distinctions between each program may not always manifest as clearly as they are described in program manuals and fidelity tools. Caregiver-mediated interventions involve two levels of implementation, that is, transfer of intervention strategies from clinician to caregiver, and subsequently from caregiver to child (Ingersoll & Wainer, 2013). In everyday practice, clinicians might not be delivering the intervention with perfect or near-perfect fidelity and may sometimes intuitively adapt based on contextual factors, family preferences, or clinical judgment. Caregivers might further modify certain strategies based on their perceived fit and ease of implementation. These adaptations may lead to a “voltage drop” in fidelity. As a result, boundaries between intervention models with varying intervention philosophies may become more blurred in such settings.

Since DSP and NDBI models differ in their use of strategies and their approach, it is not surprising that they may have different child outcomes when implemented with fidelity. A few small-scale studies have suggested that children with lower baseline levels in social communication may benefit from NDBI strategies, resulting in an increase in their expressive language targets, such as requests and responses. In contrast, children with more advanced skills may find DSP approaches more supportive, which results in an increase in their usage of comments and a few other discourse functions (see Ingersoll, 2011; Ingersoll et al., 2012; Yoder et al., 1995 for more details). We acknowledge that empirical evidence to suggest differential responses to these approaches remains limited. Future research examining how intervention philosophies and strategies are enacted in the real-world community setting might help clarify whether these nuanced distinctions translate to meaningful differences in outcomes.

Research should also explore contextual, socio-cultural, and resource adaptations made when implementing the programs included here to diverse ethno-cultural groups. Studies have shown that culturally adapted interventions yield different outcomes compared to their original versions (Zheng et al., 2021). Identifying the active ingredients of interventions and ensuring their retention during adaptation is critical for maintaining program effectiveness across different cultural contexts.

We acknowledge that the reviewers in this study are not formally trained in all the included programs; hence, a few strategies may have been missed due to a lack of nuanced understanding of the key features of the program, human error during data extraction, and limited access to program resources. Nonetheless, agreement was high between our study team and program developers/resource persons. The few disagreements that persisted were partly due to a lack of access to updated program resources to secure written confirmation. Furthermore, differences in the interpretation of intervention strategies between the team and developers contributed to some discrepancies, emphasizing the need for accurate and consistent terminology and shared understandings across the same strategies. The developers of four programs did not respond to our request to verify our data, despite multiple follow-up emails. However, given that the agreement between the reviewers and developers was on average 92% for the verified programs, it is reasonable to infer that most of the intervention strategies have been accurately captured for these four programs that lack developer confirmation with a similar degree of accuracy. Nonetheless, the absence of developer verification remains a limitation and should be interpreted accordingly.

## Conclusion

This review synthesized the aims, intervention philosophies, and targeted child skills of 18 commercially available caregiver-mediated social communication programs and explored similarities and differences of caregiver-coached strategies across 17 programs. An inventory of 70 caregiver-coached intervention strategies across the programs was identified, and data revealed semantic overlap across strategies despite functional differences in their enactment. This review is the first step toward identifying and isolating the caregiver-coached intervention elements of DSP and NDBI caregiver-mediated social communication interventions. Further research is required to determine how unique child profiles and caregiver characteristics interact with different intervention strategies and the impact of individual or clusters of strategies on child outcomes. This will allow us to gain a better understanding of which strategies or combination of strategies is most effective for whom and why, thereby enabling tailored interventions that are effective and meet each child’s needs and enhancing the scalability of caregiver-mediated programs.

## Supplemental Material

sj-pdf-1-aut-10.1177_13623613261469913 – Supplemental material for Common and Differing Elements Across Caregiver-Mediated Social Communication Programs Supporting Autistic Children: A Scoping Review With Content Analysis Supplemental material, sj-pdf-1-aut-10.1177_13623613261469913 for Common and Differing Elements Across Caregiver-Mediated Social Communication Programs Supporting Autistic Children: A Scoping Review With Content Analysis by Vipula Rajesh Kumar, Lauren Choi, Janis Oram and Amanda Binns in Autism

## Data Availability

The authors confirm that the data supporting the findings of this study are available within the article and/or its supplementary materials. Additional data may be obtained from the corresponding author upon request.
