# Supplementary material for: Common and Differing Elements Across Caregiver-Mediated Social Communication Programs Supporting Autistic Children: A Scoping Review With Content Analysis
Source: Autism. 2026 Aug 12;30(10):2443–64. doi: 10.1177/13623613261469913 (PMC13624332; doi:10.1177/13623613261469913)
Supplement: Supplementary file 1 [file sj-pdf-1-aut-10.1177_13623613261469913.pdf]

Appendix F

Strategies	Intervention																
	DIR	MTW	Inklings	PACT	RT	PFR	PLAY Project	ESDM	Project ImPACT	RIT	Social ABCs	Pathways	FPI	EMT	JASPER	WHO-CST	SCERTS
**Support pre linguistic skills/ engagement**																	
Build and sustain reciprocal back-forth interaction (e.g., through rhythm, anticipatory facial expressions, gestures, scaffolding, imitation)	Fidelity scale	Chapter 4	iBASIS manual		Mahoney & Perales (2019): Page 97, 106		Parent guide: Page 14, 22, 39	Rogers & Dawson (2010): Page 265	Ingersoll & Dvortcsak (2019) Coaching manual: Page 11	RIT Coach Lesson Guide 5,6,7		Treatment manual: Unit 3, page 2			Kasari et al. (2022): Page 336, 90		
Be sensitive and responsive to child's communication cues (e.g., look for and respond to both spoken & non-spoken communication)	Fidelity scale	Page 94, 20	iBASIS manual	Green et al. (2010) Supplementary material (SI)	Mahoney & Perales (2019): Page 55, 71, 105	Kelly et al. (2016): Page 20, 45, 46, 47	Parent guide: Page 14	Rogers & Dawson (2010): Page 267	Ingersoll & Dvortcsak (2019): Page 24		Parent manual: Page 21, 24	Treatment manual: Unit 2- page 6	Treatment manual: Page 28	Bailey et al. (2023) - Table 2	Kasari et al. (2022): Page 96, 206	Participants' guide: Page 48, 105, 262	Prizant et al. (2006): Page 26-27
Follow the child's lead	Fidelity scale	Chapter 3	iBASIS manual	Green et al. (2010): SI	Mahoney & Perales (2019): Page 30, 71	Kelly et al. (2016): Page 70, 94	Parent guide: Page 14	Rogers & Dawson (2010): Page 265-266	Ingersoll & Dvortcsak (2019): Page 24	RIT Coach Lesson Guide 3	Parent manual: Page 14, 28	Treatment manual: Unit 1,2	Treatment manual: Page 24,25	Bailey et al. (2023) - Table 2	Kasari et al. (2022): Page 90, 116	Participants' guide: Page 24, 262	Prizant et al. (2006): Page 27
Find the balance between following and leading the interaction							Parent guide: Page 43	Rogers & Dawson (2010): Page 265	Ingersoll & Dvortcsak (2019): Chapter 6	RIT Coach Lesson 5	Parent manual: Page 14; Coaching manual: Page 22	Treatment manual: Unit 8, page 2	Treatment manual: Page 39, 44		Kasari et al. (2022): Page 94, 95, 162		Prizant et al. (2006): Page 28
Use and adjust affect/animation to help child stay regulated, aroused, attentive and motivated	Fidelity scale	Page 96, 339	iBASIS manual		Mahoney & Perales (2019): Page 27, 94, 133	Kelly et al. (2016): Page 60	Parent guide: Page 24	Rogers & Dawson (2010): Page 263, 264	Ingersoll & Dvortcsak (2019): Page 36	RIT Guide: Lesson 3	Parent manual: Module 4	Treatment manual: Unit 5, page 3	Treatment manual: Page 28		Kasari et al. (2022): Page 141		Prizant et al. (2006): Page 29, 31
Observe, accept and adapt to the child's unique style of engaging (e.g., not pressuring the child to make eye contact)	Page 58, Engaging Autism	Chapter 1	iBASIS manual			Kelly et al. (2016): Page 45, 46, 47	Parent guide: Page 29										
Imitate the child	Page 312, Engaging Autism	Page 102	iBASIS manual		Mahoney & Perales (2019): Page 11	Kelly et al. (2016): Page 52	Parent guide: Page 33	Rogers & Dawson (2010): Page 106	Ingersoll & Dvortcsak (2019): Page 24	RIT Guide: Lesson 3		Treatment manual: Unit 6	Treatment manual: Page 34, 44	Bailey et al. (2023) - Table 2	Kasari et al. (2022): Chapter 9	Participants' guide: Page 80, 263	Prizant et al. (2006): Page 27
Stay face-face with the child/get down to child's level	DIR slides	Page 91	iBASIS manual		Mahoney & Perales (2019): Page 11, 94	Kelly et al. (2016): Page 56	Parent guide: Page 36	Rogers & Dawson (2010): Page 104	Ingersoll & Dvortcsak (2019): Page 24	RIT Guide: Lesson 3	Parent manual: Page 35, 45	Treatment manual: unit 2, page 7		Bailey et al. (2023) - Table 2	Kasari et al. (2022): Page 124	Participants' guide: Page 26, 262	Prizant et al. (2006): Page 29
Engage in turn taking (e.g., use anticipatory phrases to warn the child, make adult's turns short at first, use adult's turn to model new ways to play with the object)	DIR Slides	Page 59, Chapter 4- page 112			Mahoney & Perales (2019): Page 16		Parent guide: Page 43	Rogers & Dawson (2010): Page 107, 265	Ingersoll & Dvortcsak (2019): Page 57			Treatment manual: Unit 8	Treatment manual: Page 26, 30	Bailey et al. (2023) - Table 2	Kasari et al. (2022): Page 96, 210		Prizant et al. (2006): Page 28, 34
Encourage eye contact to mand for objects (e.g., get into the child's line of sight through prompts/ reinforcement/ waiting/ blocking)								Rogers & Dawson (2010): Page 165, 158, 159				Treatment manual: Unit 3, 7					

Strategies	Intervention																
	**DIR**	**MTW**	**Inklings**	**PACT**	**RT**	**PFR**	**PLAY Project**	**ESDM**	**Project ImPACT**	**RIT**	**Social ABCs**	**Pathways**	**FPI**	**EMT**	**JASPER**	**WHO-CST**	**SCERTS**
**Facilitate learning of new skills**																	
Provide behavioral teaching trials (e.g., ABC, trials, repetition)								Rogers & Dawson (2010): Page 262	Ingersoll & Dvortcsak (2019) Coaching manual: Page 127	RIT Coach: Lesson 7	Parent manual: Module 1	Treatment manual: unit 1, page 5; Unit 7		Kaiser & Hampton (2017) - Page 91	Kasari et al. (2022): Chapter 5		
Provide instructional teaching techniques (e.g., prompting, fading, shaping, chaining) to teach communication, play, etc.								Rogers & Dawson (2010): Page 262	Ingersoll & Dvortcsak (2019): Page 68	RIT Coach Lesson 8	Parent manual: Module 6	Treatment manual: unit 4, page 7	Treatment manual: Page 11	Kaiser & Hampton (2017) - Page 91	Kasari et al. (2022): Page 142		Prizant et al. (2006): Page 19
Provide extrinsic reinforcement (e.g., social reinforcers, edible reinforcers, etc.) any time the child's response matches the caregiver's expectation (for a "good" response)		Page 45, 102					Parent guide: Page 38	Rogers & Dawson (2010): Page 182, 132	Ingersoll & Dvortcsak (2019) Page 86	RIT Coach Lesson Guide 6		Treatment manual: Unit 4	Treatment manual: Page 39	Parent handbook 1 - Page 55		Participants' guide: Page 59, 262	
Provide natural reinforcement (e.g., desired object given to child after the child requests for it) any time the child's response matches the caregiver's expectation (for a "good" response)		Chapter 3						Rogers & Dawson (2010): Page 266	Ingersoll & Dvortcsak (2019): Page 72	RIT Coach Lesson Guide 6	Parent manual: Page 19	Treatment manual: Unit 4		Davison et al. (2019)	Kasari et al. (2022): Page 140	Participants' guide: Page 48	
Provide reinforcement for all attempts made by the child irrespective of whether child's response matches caregiver's expectation		Page 94				Kelly et al. (2016): Page 48, 97			Ingersoll & Dvortcsak (2019): Page 72							Participants' guide: Page 155	
Establish joint activity structure (beginning-middle-end)/establish routines		Page 57, 258	iBASIS manual	Green et al. (2010): SI	Mahoney & Perales (2019): Page 47, 144	Kelly et al. (2016): Page 56, 68	Parent guide: Page 40	Rogers & Dawson (2010): Page 269	Ingersoll & Dvortcsak (2019): Page 12	RIT Coach Lesson 5	Parent manual: Page 54	Treatment manual: Unit 1, page 7; unit 5, page 8	Treatment manual: Page 15-19	Parent handbook 1 - Page 55	Kasari et al. (2022): Chapter 5	Participants' guide: Page 263	Prizant et al. (2006): Page 19, 34
Elaborate joint activity structure/routines by building on child's play/communication		Page 163-168		Green et al. (2010): SI	Mahoney & Perales (2019)	Kelly et al. (2016)		Rogers & Dawson (2010)			Parent manual: Page 75-77		Treatment manual	Bailey et al. (2023) - Table 2	Kasari et al. (2022): Chapter 5	Participants' guide: Page 240, 266	Prizant et al. (2006)
Use time delay strategies (e.g., wait for child to react after providing an opportunity to respond, take one turn and wait)	Fidelity scale	Page 88, 275	iBASIS manual	Green et al. (2010): SI	Mahoney & Perales (2019): Page 16		Parent guide: Page 37, 38	Rogers & Dawson (2010): Page 157, 164, 178	Ingersoll & Dvortcsak (2019): Page 24	RIT Coach Lesson Guide 6	Coaching manual: 86	Treatment manual: Unit 1, page 10; Unit 2, page 7	Treatment manual: Page 35	Parent handbook 1 - Page 24	Kasari et al. (2022): Page 96, 160	Participants' guide: Page 108, 263	Prizant et al. (2006): Page 28
Use cognitive scaffolding (e.g., offering hints, visual supports; playful obstruction, signaling with gestures, reducing barriers to success, structuring the environment to ensure the child can meaningfully explore, encouraging self-exploration)	Fidelity scale	Page 114, 129			Mahoney & Perales (2019): Page 62??	Kelly et al. (2016): Page 48, 70	Parent guide: Page 37, 41	Rogers & Dawson (2010): Page 157	Ingersoll & Dvortcsak (2019) Coaching manual: Page 11			Treatment manual: Unit 8, 9			Kasari et al. (2022): Chapter 7, 8	Participants' guide: Session 6 chapter	Prizant et al. (2006): Page 36
Provide hand-over-hand guidance/gentle physical guidance when child is unable to provide a correct/target response		Page 115, 384							Ingersoll & Dvortcsak (2019): Page 68	RIT Coach Lesson Guide 6		Treatment manual: Unit 4, page 7; Unit 7, page 10			Kasari et al. (2022): Page 142	Participants' guide: Page 154	

Strategies	Intervention																
	**DIR**	**MTW**	**Inklings**	**PACT**	**RT**	**PFR**	**PLAY Project**	**ESDM**	**Project ImPACT**	**RIT**	**Social ABCs**	**Pathways**	**FPI**	**EMT**	**JASPER**	**WHO-CST**	**SCERTS**
**To support expressive communication and comprehension**																	
Talk about what the child is seeing, hearing or doing (i.e., commenting)	DIR Slides	Page 61, 93-94	iBASIS manual	Green et al. (2010): SI	Mahoney & Perales (2019): Page 30, 121	Kelly et al. (2016): Page 47, 48, 79, 95	Parent guide: Page 33	Rogers & Dawson (2010): Page 105	Ingersoll & Dvortcsak (2019): Page 36	RIT Guide: Lesson 3		Treatment manual: Unit 10, page 8	Treatment manual: Page 39	Parent handbook 1 - Page 19	Kasari et al. (2022): Page 103	Participants' guide: Page 109, 263	Prizant et al. (2006): Page 27
Avoid asking a lot of questions or making directive commands	Fidelity scale	Page 126	iBASIS manual	Green et al. (2010): SI	Mahoney & Perales (2019): Page 30, 147		Parent guide: Page 33	Rogers & Dawson (2010): Page 268	Ingersoll & Dvortcsak (2019): Page 24, 36	RIT Guide: Lesson 3, 4	Coaching manual: Page 31	Treatment manual: Unit 1, page 11, Unit 2-page 7	Treatment manual: Page 40; Parent workbook: Page 43	Bailey et al. (2023) - Table 2	Kasari et al. (2022): Page 94	Participants' guide: Page 108	Prizant et al. (2006): Page 33
Model language that is within the child's perceived zone of proximal development	Fidelity scale	Page 116		Green et al. (2010): SI	Mahoney & Perales (2019): Page 71, 105		Parent guide: Page 44	Rogers & Dawson (2010): Page 268-269	Ingersoll & Dvortcsak (2019): Page 43	RIT Coach: Lesson 4		Treatment manual: Unit 10, page 8-9	Treatment manual: Page 34	Kaiser & Hampton (2017) - Page 91	Kasari et al. (2022): 101	Participants' guide: Page 107, 263, 264	Prizant et al. (2006): Page 30
Say and repeat familiar words/phrases while interacting with the child (i.e., modelling, focused stimulation)		Page 198, 259		Green et al. (2010): SI			Parent guide: Page 36	Rogers & Dawson (2010): Page 174	Ingersoll & Dvortcsak (2019): Page 45	RIT Coach Guide: Lesson 4		Treatment manual: Unit 10, page 10					
Provide multiple and varied communication opportunities in an activity		Page 394	iBASIS manual	Green et al. (2010): SI			Parent guide: Page 33	Rogers & Dawson (2010): Page 140, 267	Ingersoll & Dvortcsak (2019)		Parent manual: Page 35, 46	Treatment manual: Unit 9,10					Prizant et al. (2006): Page 34,35
Speak slowly and emphasise target/key words while interacting with the child		Page 196					Parent guide: Page 36		Ingersoll & Dvortcsak (2019): Page 36	RIT Coach Lesson Guide 4		Treatment manual: Last 2 pages of Unit 10	Treatment manual: Page 39				
Ensure child is interested and paying attention, then connect what the child sees/wants with hearing its label, trying to say it and getting it											Parent manual: Page 17						
Clearly point to or show objects while verbally labeling to make the object names more salient.		Page 93			Mahoney & Perales (2019): Page 136		Parent guide: Page 39	Rogers & Dawson (2010): Page 166						Bailey et al. (2023) - Table 2	Kasari et al. (2022): Page 209		
Balance the adult's use of communicative functions								Rogers & Dawson (2010): Page 269									Prizant et al. (2006): Page 31
Provide comments that are contextual, simple, and do not contain words that are the target goals for the child (i.e., model prompts)											Coaching manual: Page 28						
Use contextual/environmental cues to amplify meaning of targeted aspects of language	Fidelity scale								Ingersoll & Dvortcsak (2019): Page 45					Parent handbook 1 - Page 9			
Provide explicit prompts for communication (e.g., prompt target word/action)		Page 98						Rogers & Dawson (2010): Page 159	Ingersoll & Dvortcsak (2019): Page 68, 74	RIT Coach Lesson Guide 8	Parent manual: Page 15	Treatment manual: unit 9, page 4	Treatment manual: Page 11	Parent handbook 1 - Page 42	Kasari et al. (2022): Page 142, 231		

Strategies	Intervention																
	**DIR**	**MTW**	**Inklings**	**PACT**	**RT**	**PFR**	**PLAY Project**	**ESDM**	**Project ImPACT**	**RIT**	**Social ABCs**	**Pathways**	**FPI**	**EMT**	**JASPER**	**WHO-CST**	**SCERTS**
Use communicative scaffolding (e.g., provide choices, make deliberate mistakes for the child to correct)	Fidelity scale	Page 275		Green et al. (2010): SI	Mahoney & Perales (2019): Page 74, 192	Kelly et al. (2016): Page 100	Parent guide: Page 36	Rogers & Dawson (2010): Page 268	Ingersoll & Dvortcsak (2019): Page 52			Treatment manual: Unit 8, page 4; Unit 10	Treatment manual: Page 44	Parent handbook 1 - Page 42	Kasari et al. (2022): Page 223-228	Participants' guide: Page 24, 131, 264	Prizant et al. (2006): Page 19, 28
Entice the child to initiate (e.g., by setting up/creating opportunities for communication; communicative temptations)	Page 89, Engaging Autism	Page 59, 68-74		Green et al. (2010): SI		Kelly et al. (2016)	Parent guide: Page 22	Rogers & Dawson (2010): Page 157, 163	Ingersoll & Dvortcsak (2019): Page 52, 61		Parent manual: Module 1	Treatment manual: Unit 2, page 2	Treatment manual: Page 44	Parent handbook 1 - Page 43	Kasari et al. (2022): Page 220	Participants' guide: Page 128, 131, 264	Prizant et al. (2006): Page 20
Respond to the child's intention even if the child's word choices/pronunciations may not be the expected response	Language Development Slides/Videos	Page 94	iBASIS manual		Mahoney & Perales (2019): Page 56, 117	Kelly et al. (2016): Page 78	Parent guide: Method 1, page 14		Ingersoll & Dvortcsak (2019): Page 28						Kasari et al. (2022): Page 208	Participants' guide: Page 106, 263	Prizant et al. (2006): Page 27, 28
Restate/recast the child's utterance	DIR 103 slides	Page 102		Green et al. (2010): SI	Mahoney & Perales (2019): Page 11	Kelly et al. (2016): Page 60	Parent guide: Page 35	Rogers & Dawson (2010): Page 267	Ingersoll & Dvortcsak (2019): Page 24	RIT Coach Lesson 3	Parent manual: Page 29	Treatment manual: Unit 6, page 6			Kasari et al. (2022): Page 206	Participants' guide: Page 130, 264	Prizant et al. (2006): Page 27
Expand the child's utterance				Green et al. (2010): SI	Mahoney & Perales (2019): Page 59		Parent guide: Page 40	Rogers & Dawson (2010): Page 267	Ingersoll & Dvortcsak (2019): Page 36, 46	RIT Coach: Lesson 4	Parent manual: Page 86	Treatment manual: Unit 10, page 8		Bailey et al. (2023) - Table 2	Kasari et al. (2022): Page 206	Participants' guide: Page 130, 264	Prizant et al. (2006): Page 27
Utilize AAC to make it easier for the child to interact	DIR 201 Slides	Page 213-254						Rogers & Dawson (2010): Page 133				Treatment manual: Unit 10, page 4		Kaiser & Hampton (2017) - Page 89	Kasari et al. (2022): Chapter 14		Prizant et al. (2006): Page 35
**To support play development**																	
Model play to the child based on the child's functional developmental level		Chapter 11			Mahoney & Perales (2019): Page 71, 77-	Kelly et al. (2016): Page 70, 96	Parent guide: page 44, 51	Rogers & Dawson (2010): Page 146	Ingersoll & Dvortcsak (2019): Page 59	RIT Coach Lesson 5	Coaching manual: Page 74		Treatment manual: Page 34	Parent handbook 1 - Page 19, 59	Kasari et al. (2022): Chapter 9	Participants' guide: Page 57	Prizant et al. (2006): Page 31
Provide multiple and varied play opportunities in an activity	202 DIR Sldies					Kelly et al. (2016)	Parent guide: Play 33, 36	Rogers & Dawson (2010): Page 140	Ingersoll & Dvortcsak (2019): Page 83	RIT Coach Lesson 5	Parent manual: Page 51				Kasari et al. (2022): Page 101	Participants' guide: Page 57	
Use toys creatively (depart from conventional ways of using the toys and adapt to the child's way of playing)	DIR 101 slides		iBASIS manual			Kelly et al. (2016): Page 70	Parent guide: Page 29			RIT Coach Lesson 3	Parent manual: Page 51; Coaching manual: Page 74		Treatment manual: Page 33, 34	Parent handbook 1 - Page 18			
Let child initiate the play idea and through interaction (dramatization in roles), elaborate with expanding themes and range of emotions (closeness, assertiveness, fear, anger, jealousy, aggression, etc.) which the child can explore and express safely	202 DIR Slides						Parent guide: Page 26	Rogers & Dawson (2010): Page 152-153									
Expand the child's play interests/play	Page 286, Engaging Autism	Chapter 11	iBASIS manual	Green et al. (2010): SI	Mahoney & Perales (2019): Page 19, 59	Kelly et al. (2016): Page 70, 95	Parent guide: Page 33	Rogers & Dawson (2010): Page 107, 140	Ingersoll & Dvortcsak (2019): Page 68, 88		Coaching manual: Page 75	Treatment manual: Unit 5,8,9	Treatment manual: Page 33	Bailey et al. (2023) - Table 2	Kasari et al. (2022): Chapter 11	Participants' guide: Page 79	Prizant et al. (2006): Page 30
Model appropriate/conventional play when the child uses inappropriate/unconventional play		Page 398							Ingersoll & Dvortcsak (2019): Page 32		Coaching manual: Page 74	Treatment manual: Unit 5, page 4; Unit 8, page 5			Kasari et al. (2022): Page 159		Prizant et al. (2006): Page 31

Strategies	Intervention																
	DIR	MTW	Inklings	PACT	RT	PFR	PLAY Project	ESDM	Project ImPACT	RIT	Social ABCs	Pathways	FPI	EMT	JASPER	WHO-CST	SCERTS
**To provide supports through environmental arrangement**																	
Create clearly defined spaces & materials and limit distractions and clutter	Fidelity scale				Mahoney & Perales (2019): Page 4	Kelly et al. (2016): Page 99	Parent guide: Page 52	Rogers & Dawson (2010): Page 103, 129, 187	Ingersoll & Dvortcsak (2019): Page 14	RIT Guide: Lesson 2		Treatment manual: Unit 2, page 4	Treatment manual: Page 17	Bailey et al. (2023) - Table 2	Kasari et al. (2022): Chapter 7	Participants' guide: Page 23, 181, 262	Prizant et al. (2006): Page 36
Support transition between activities (e.g., provide warnings)						Kelly et al. (2016): Page 78		Rogers & Dawson (2010): Page 270	Ingersoll & Dvortcsak (2019): Page 125		Parent manual: Page 60		Treatment manual: Page 18	Parent handbook 1 - Page 56	Kasari et al. (2022): Page 134-138	Participants' guide: Page 181, 265	Prizant et al. (2006): Page 32
Provide assistive visual or organizational supports (when appropriate)	Page 293, Engaging Autism	Page 213-254					Parent guide: Page 45	Rogers & Dawson (2010): Page 133, 202						Teaching play - slide 19	Kasari et al. (2022): Chapter 8	Participants' guide: Page 266	Prizant et al. (2006): Page 23, 35
Use sensorimotor and/or motor supports	Fidelity scale	Page 380	iBASIS manual			Kelly et al. (2016): Page 61	Parent guide: Page 35	Rogers & Dawson (2010): Page 110-114									Prizant et al. (2006): Page 36
**To provide interpersonal/relational supports**																	
Be physically available and interactive	DIR 101 slides		iBASIS manual		Mahoney & Perales (2019): Page 160	Kelly et al. (2016): Page 47, 56	Parent guide: Page 35	Rogers & Dawson (2010): page 105			Coaching manual: Page 72						
Use a range of affective cues and responses (e.g., intonation, facial expressions, gestures, non-verbal communication)	Fidelity scale	Page 17, 242	iBASIS manual		Mahoney & Perales (2019): Page 88, 133	Kelly et al. (2016): Page 60, 80	Parent guide: Page 22, 24, 39	Rogers & Dawson (2010): Page 160	Ingersoll & Dvortcsak (2019): Page 37, 38			Treatment manual: Unit 5, page 3,4			Kasari et al. (2022): Page 280		Prizant et al. (2006): Page 29
Use positive affect/positive emotion sharing					Mahoney & Perales (2019): Page 88, 164	Kelly et al. (2016): Page 47, 52, 78, 79	Parent guide: Page 19, 21	Rogers & Dawson (2010): Page 266	Ingersoll & Dvortcsak (2019) Coaching manual: Page 384		Parent manual: Page 20, 30, 32	Treatment manual: Unit 5, page 4	Treatment manual: Page 18, 39, 40		Kasari et al. (2022): Page 140, 141, 209	Participants' guide: Page 49	
Accept, empathise, and validate all the child's feelings, even negative ones	Fidelity scale		iBASIS manual		Mahoney & Perales (2019): Page 163	Kelly et al. (2016): Page 45, 46, 47	Parent guide: Page 48	Rogers & Dawson (2010): Page 267									
Recognize and label the child's feelings		Page 242	iBASIS manual		Mahoney & Perales (2019): Page 144, 177	Kelly et al. (2016): Page 45, 46, 48	Parent guide: Page 47	Rogers & Dawson (2010): Page 162	Ingersoll & Dvortcsak (2019) Coaching manual: Page 310								
Use touch, voice, body language and facial expressions to help child understand their feelings		Page 242	iBASIS manual		Mahoney & Perales (2019): Page 179	Kelly et al. (2016): Page 47, 80	Parent guide: Page 47	Rogers & Dawson (2010): Page 162									
Teach child acceptable ways of expressing strong feelings (e.g., provide concrete suggestions for what the child can say or do to express their strong feeling)		Page 110			Mahoney & MacDonald (2007)	Kelly et al. (2016): Page 48, 81											

Strategies	Intervention																
	**DIR**	**MTW**	**Inklings**	**PACT**	**RT**	**PFR**	**PLAY Project**	**ESDM**	**Project ImPACT**	**RIT**	**Social ABCs**	**Pathways**	**FPI**	**EMT**	**JASPER**	**WHO-CST**	**SCERTS**
**To prevent challenging or interfering behavior**																	
Identify trigger(s) that may underlie the child's interfering behaviors and employ strategies to alleviate the distress (e.g., avoid the trigger, provide warnings before the trigger occurs, modify the environment to reduce the impact of the trigger)			iBASIS manual			Kelly et al. (2016): Page 58		Rogers & Dawson (2010): Page 264	Ingersoll & Dvortcsak (2019): Page 117		Parent manual: Page 59	Treatment manual: unit 11	Treatment manual: Page 18		Kasari et al. (2022): Page 278	Participants' guide: Page 180, 265	
Give clear instructions and set expectations to prevent challenging behaviors					Mahoney & Perales (2019): Page 200	Kelly et al. (2016): Page 48, 87			Ingersoll & Dvortcsak (2019): Page 117, 124					Parent handbook 1 - Page 56	Kasari et al. (2022): Page 279, 282	Participants' guide: Page 202, 265	
Catch the child exhibiting 'good' behavior and praise them for it						Kelly et al. (2016): Page 47	Parent guide: Page 59					Treatment manual: Unit 11, page 7		Parent handbook 1 - Page 55	Kasari et al. (2022): Page 286	Participants' guide: Page 177, 265	
**To support child in times of distress or when expressing sensory needs**																	
Redirect away from interfering behaviours	Page 87, Engaging Autism		iBASIS manual		Mahoney & Perales (2019): Page 47	Kelly et al. (2016): Page 47, 101		Rogers & Dawson (2010): Page 264	Ingersoll & Dvortcsak (2019): Page 73		Parent manual: Page 65	Treatment manual: Unit 11, page 6	Treatment manual: Page 25	Parent handbook 1 - Page 58	Kasari et al. (2022): Page 282		
Ignore the 'challenging' behavior					Mahoney & Perales (2019): Page 47		Parent guide: Page 60				Parent manual: Page 65	Treatment manual: Unit 11, page 7		Parent handbook 1 - Page 57	Kasari et al. (2022): Page 283	Participants' guide: Page 201, 265	
Pause and take a few breaths, use self-talk, and repeat a key word that calms the caregiver during moments of disruption in caregiver-child relationship						Kelly et al. (2016): Page 64											
Respond sensitively to the child's distress by using gentle touch, tone of voice, facial expressions, rocking, holding, singing, and/or offering comfort to co-regulate the child	Page 59, 362, Engaging Autism		iBASIS manual		Mahoney & Perales (2019): Page 167, 181	Kelly et al. (2016): Page 47, 52, 61	Parent guide: Page 19				Parent manual: Module 4						
Facilitate experiences for the child that satisfy sensory needs akin to their repetitive/self-stimulatory behavior	Page 313, 317, 320, Engaging Autism	Page 380	iBASIS manual				Parent guide: Page 29, 33					Treatment manual: Unit 11, page 6				Participants' guide: Page 202, 218	
When the child is distressed/ dysregulated, change the activity being done, lessen the stimulation, or take a break	Fidelity scale					Kelly et al. (2016): Page 61		Rogers & Dawson (2010): Page 263, 266			Coaching manual: Page 66		Treatment manual: Page 36		Kasari et al. (2022): Page 280		Prizant et al. (2006): Page 28
Encourage self-soothing (e.g., offer blankets, favourite stuffed animal, something to suck on)						Kelly et al. (2016): Page 47, 61, 64					Coaching manual: Page 41						
Use language to re-interpret and describe the child's actions in a way that assumes positive intention (e.g., if the child throws a toy car in the corner, the parent may say "all done car)			iBASIS manual				Parent guide: Page 35						Treatment manual: Page 41				Prizant et al. (2006): Page 29
Respond to challenging behavior that aims to get access by teaching the child to use communication skills							Parent guide: Page 61		Ingersoll & Dvortcsak (2019): Page 131						Kasari et al. (2022): Page 280, 282	Participants' guide: Page 201, 266	
Model appropriate/conventional behavior when the child uses inappropriate/unconventional behavior if the child is regulated enough to benefit from models (e.g., model appropriate mouthing and biting using a chewy when the child is chewing on their clothes to self-regulate arousal)								Rogers & Dawson (2010): Page 123	Ingersoll & Dvortcsak (2019): Page 32	RIT Guide: Lesson 3					Kasari et al. (2022): Page 163, 310	Participants' guide: Page 202, 218, 266	Prizant et al. (2006): Page 31
Use reinforcement strategies to teach adaptive/conventional behaviors (e.g., positive behavior supports)								Rogers & Dawson (2010): Page 120	Ingersoll & Dvortcsak (2019): Page 117			Treatment manual: Unit 11					

Strategies	Intervention																
	**DIR**	**MTW**	**Inklings**	**PACT**	**RT**	**PFR**	**PLAY Project**	**ESDM**	**Project ImPACT**	**RIT**	**Social ABCs**	**Pathways**	**FPI**	**EMT**	**JASPER**	**WHO-CST**	**SCERTS**
Provide discipline with choices/limits/responsive guidance	Page 338, Engaging Autism				Mahoney & Perales (2019): Page 168	Kelly et al. (2016): Page 47, 100	Parent guide: Page 60		Ingersoll & Dvortcsak (2019): Page 117								
Provide a 3-step follow through when the child exhibits an inappropriate behavior														Parent handbook 1 - Page 57			
